# Efficacy of romosozumab as sequential therapy for bisphosphonates in glucocorticoid-induced osteoporosis

**DOI:** 10.1093/jbmrpl/ziag116

**Published:** 2026-07-21

**Authors:** Mai Kawazoe, Sei Muraoka, Zento Yamada, Wataru Hirose, Eri Watanabe, Junko Nishio, Toshihiro Nanki

**Affiliations:** Division of Rheumatology, Department of Internal Medicine, Toho University School of Medicine, Tokyo 143-8541, Japan; Division of Rheumatology, Department of Internal Medicine, Toho University School of Medicine, Tokyo 143-8541, Japan; Division of Rheumatology, Department of Internal Medicine, Toho University School of Medicine, Tokyo 143-8541, Japan; Hirose Clinic of Rheumatology, Saitama 359-1111, Japan; Division of Rheumatology, Department of Internal Medicine, Toho University School of Medicine, Tokyo 143-8541, Japan; Division of Rheumatology, Department of Internal Medicine, Toho University School of Medicine, Tokyo 143-8541, Japan; Division of Rheumatology, Department of Internal Medicine, Toho University School of Medicine, Tokyo 143-8541, Japan

**Keywords:** bisphosphonates, bone mineral density, glucocorticoid-induced osteoporosis, romosozumab, sclerostin, Wnt signaling

## Abstract

Glucocorticoids (GCs) affect the Wnt signaling pathway, which is a key mechanism underlying GC-induced osteoporosis (GIOP). The efficacy of romosozumab (ROMO), an antibody targeting sclerostin—an inhibitor of Wnt signaling—in treating GIOP remains unclear, as does its effectiveness when used sequentially after bisphosphonates (BP). In this prospective observational study, we aimed to evaluate the efficacy of ROMO as a sequential therapy following BP in patients with GIOP. Patients with rheumatic diseases receiving BP and GCs equivalent to 5 mg/d or more of prednisolone for at least 6 mo were enrolled and either switched from BP to ROMO (ROMO group) or continued on BP (BP group). BMD of the LS (L2**-**4), FN, and TH were measured every 6 mo, and bone turnover markers were assessed every 3 mo. Six patients in the ROMO group and 22 in the BP group were analyzed. Patients in the ROMO group were significantly older (78.5 vs 64.5 yr), had a higher history of vertebral fractures, had lower baseline BMD of the FN and TH, and tended to receive higher GC dose (8.0 mg vs 5.0 mg) compared with the BP group. The median (25th**-**75th percentile) BMD percent changes at 12 mo were numerically higher in the ROMO group compared to the BP group (LS 2.6 [0.8**-**3.4] % vs 1.6 [−1.9−4.3] %; FN 1.4 [−0.5−5.4] % vs 1.0 [−4.7−5.6] %; TH: 1.1 [−7.6−6.5] % vs −1.8 [−6.4−1.8] %), although their differences were not statistically significant. One new vertebral fracture was observed in the BP group, and 1 patient in the ROMO group experienced a cerebral infarction. These findings suggest that ROMO may be an effective sequential therapy for BP in patients with GIOP.

## Introduction

Glucocorticoids (GCs) are used to treat various conditions, including rheumatic diseases, and they improve treatment outcomes. However, one of their side effects is GC-induced osteoporosis (GIOP), which is the most common form of secondary osteoporosis.^1^ Glucocorticoids cause bone loss and fragility by promoting bone resorption and suppressing bone formation. Approximately 6%**-**12% of BMD is lost within the first year of GC therapy, followed by a further decline of around 3% per yr.^2^ Fracture rates increase by 50% with oral prednisolone (PSL) doses of 5 mg/d or higher for 3 mo or longer, with fractures occurring in 30%**-**50% of long-term users.^3^ Furthermore, GC exposure primarily causes trabecular bone loss, increasing the risk of vertebral fractures. Previous epidemiological studies report a 2.59-fold higher risk with PSL doses of 2.5 mg/d or more, but less than 7.5 mg/d, compared to the non-exposed group.^4^

The Wnt signaling pathway is considered to be one of the mechanisms underlying GIOP and promotes bone formation while inhibiting bone resorption. This pathway is inactivated by negative regulators of Wnt signaling, including sclerostin and Dickkopf-1 (Dkk-1). We previously demonstrated that GC therapy increases serum sclerostin and decreases Wnt3a, a ligand for the Wnt signaling pathway.^5^^,^^6^ These findings suggest that inhibiting sclerostin could be an effective treatment for GIOP.

Romosozumab (ROMO), a humanized anti-sclerostin monoclonal antibody, has been shown to promote bone formation and inhibit bone resorption. Large randomized controlled trials (RCTs) in patients with PMO demonstrated that, compared to placebo or bisphosphonates (BP), ROMO significantly increased the BMD of the LS, FN and TH after 12 mo, and reduced the incidence of both vertebral and non-vertebral fractures.^7^^,^^8^

The first-line treatment for GIOP, as stated in the prior Japanese Society for Bone and Mineral Research’s Guidelines for the Management and Treatment of GIOP (2014 Revised Edition),^9^ is BP, an antiresorptive agent. Consequently, BPs are administered to many patients with GIOP. However, in our daily clinical practice of treating patients with rheumatic diseases, we frequently encounter patients with GIOP who experience new or multiple vertebral fractures despite receiving BP therapy.

We recently reported that ROMO increases BMD of the LS, FN and TH to a greater extent than BP do in patients newly initiating moderate to high dose GC therapy.^10^ However, there is little information on the efficacy and safety of using ROMO sequentially in patients with GIOP following long-term BP exposure, which is an issue that requires urgent resolution. Therefore, this study was conducted to clarify the efficacy and safety of ROMO as sequential therapy following BP in patients with GIOP, compared with continued BP administration.

## Materials and methods

### Study design and participants

We conducted a prospective observational study beginning in July 2019. Patients with rheumatic diseases who were receiving GCs equivalent to 5 mg or more of PSL per day and BP for 6 mo or longer, were included. The following patients were excluded: those with contraindications to ROMO; those with conditions requiring caution for ROMO use (eg, a history of cardiovascular or cerebrovascular events within the past year); those with active infections or malignancies; those under 20 yr of age; and those wishing to become pregnant. Decisions regarding osteoporosis treatment (switching from BP to ROMO or continuing BP) were made by the attending physician for each patient. Patients who switched from BP to ROMO were classified as the ROMO group, while those who continued with BP were classified as the BP group.

The primary endpoint was the changes in LS BMD at 12 mo. Secondary endpoints included changes in the BMD of the FN and TH at 12 mo, changes in bone turnover markers over 12 mo, incidence of new fractures, and adverse events.

### Measurement of BMD

DXA was performed every 6 mo to evaluate the BMD of the LS (L2**-**4), FN, and TH. Discovery A (Hologic, Waltham, MA, USA) was used for measurements, automatically calculating BMD from bone area (cm^2^), and BMC (g), with the results expressed in g/cm^2^.

### Biochemical markers

Fasting morning blood samples were collected at baseline and at 3, 6, 9, and 12 mo after the start of the study to assess bone turnover markers. A subset of the serum samples was promptly frozen at −80 °C and preserved until analysis via ELISA. Serum concentrations of bone formation markers, including total P1NP and osteocalcin (OC), were quantified using electrochemiluminescence immunoassays. Serum tartrate-resistant acid phosphatase 5b (TRACP-5b), a bone resorption marker, was measured using enzyme immunoassays. Urinary pentosidine, which serves as a bone matrix marker, was assessed using HPLC. Serum 25OHD concentrations were also measured using chemiluminescent enzyme immunoassay. Additional biomarkers, including serum sclerostin, Dkk-1, RANKL, and osteoprotegerin (OPG) (R&D Systems, Minneapolis, MN, USA) and Wnt3a (ABclonal, Woburn, MA, USA), were quantified using ELISA kits in accordance with the manufacturers’ instructions.

### Statistical analysis

The baseline characteristics of patients within the safety analysis set, defined as those who received at least 1 dose of the investigational drug, were expressed using medians with interquartile ranges for continuous variables, and *n* (%) for categorical variables. Differences between the 2 groups for numerical data were assessed using the Kruskal-Wallis test. Changes in BMD and bone turnover markers within each group were evaluated as percent changes from baseline using the full analysis set (FAS). Temporal changes within each group were examined using Dunn’s multiple comparison test, while differences between baseline and each subsequent time point across groups were performed using Bonferroni’s multiple comparison test. In addition, the percent changes in BMD from baseline to 12 mo were compared between the ROMO and BP groups using a linear regression model adjusted for age. Results are presented as regression coefficients (*β*) with corresponding 95% CIs. A *p*-value of <.05 was considered statistically significant. Multivariable analyses were performed using R version 4.4.0 (R Core Team 2024, Vienna, Austria), while other statistical procedures were conducted using Prism version 9.0 (GraphPad Software, San Diego, CA, USA).

### Ethical statement

This study was approved by the Ethics Committee of Toho University Omori Medical Center (approval number; M18256_M24215 23 064), and it complied with the 1964 Declaration of Helsinki and its later amendments and Ethical Guidelines for Medical and Health Research Involving Human Subjects by the Ministries of Education, Culture, Sports, Science and Technology and Health, Labour and Welfare of the Japanese Government. All participants who were included in this study gave their written informed consent.

## Results

### Patient disposition and characteristics

A total of 30 patients were enrolled: 6 in the ROMO group and 24 in the BP group. However, 2 patients in the BP group withdrew their consent and were therefore excluded from all analyses. The baseline characteristics of patients are summarized in Table 1. The age of the ROMO group was significantly higher than that of the BP group (78.5 yr vs 64.5 yr; *p* = .001). There was no significant difference in the male-to-female ratio between the 2 groups. Although not statistically significant, the daily GC dose tended to be higher in the ROMO group (8.0 mg vs 5.0 mg; *p* = .19), and the duration of BP administration before the start of the study tended to be longer in the BP group (25.0 mo vs 58.5 mo; *p* = .47). The history of vertebral fractures prior to the start of the study was significantly more common in the ROMO group. Baseline BMD of the LS was comparable between the 2 groups; however, the ROMO group showed lower BMD of the FN and TH. Urinary pentosidine levels were significantly higher in the ROMO group (*p* < .0001). Baseline values for other bone turnover markers and molecules regulating bone metabolism were similar between the 2 groups.

**Table 1:** Demographics and clinical data at baseline of the study population.

	**ROMO group (*n* = 6)**	**BP group (*n* = 22)**	***p* value**
**Age (years)**	78.5 [74.5-82.3]	64.5 [49.8-71.5]	.001
**Male/female**	0/6	3/19	.56
**Postmenopausal women (%)**	6 (100)	16 (84.2)	.55
**BMI (kg/m** ^**2**^**)**	19.8 [16.5-21.8]	20.9 [19.0–22.9]	.17
**Daily prednisolone dose (mg)**	8.0 [5.0-14.6]	5.0 [5.0–6.3]	.19
**Duration of bisphosphonates use (months)**	25.0 [6.0-170.8]	58.5 [17.8-110.3]	.47
**Prior vertebral fracture, no. (%)**	2 (33.3)	0 (0)	.04
**Prior nonvertebral fracture, no. (%)**	0 (0)	1 (4.2)	>.99
**Diagnosis, no. (%)**			
** Vasculitis syndrome**	3 (50.0)	5 (20.8)	
** Systemic lupus erythematosus**	1 (16.7)	6 (25.0)	
** Systemic sclerosis**	1 (16.7)	1 (4.2)	
** Rheumatoid arthritis**	1 (16.7)	0 (0)	
** Adult-onset Still’s disease**	0 (0)	2 (8.3)	
** IgG4 related disease**	0 (0)	2 (8.3)	
** Polymyositis/Dermatomyositis**	0 (0)	2 (8.3)	
** Behçet’s disease**	0 (0)	1 (4.2)	
** Sjögren’s disease**	0 (0)	1 (4.2)	
** Mixed connective tissue disease**	0 (0)	1 (4.2)	
** Relapsing polychondritis**	0 (0)	1 (4.2)	
**Serum markers**			
** eGFR (mL/min/1.73m** ^**2**^**)**	71.4 [64.6-78.0]	64.6 [56.4-75.5]	.37
** 25OHD (ng/mL)**	15.5 [8.2–18.3]	15.9 [12.7-17.8]	.59
** P1NP (μg/L)**	19.0 [13.6-70.8]	23.6 [15.7-34.6]	.97
** OC (ng/mL)**	4.8 [3.5–6.6]	6.8 [5.7-11.6]	.05
** TRACP-5b (mU/dL)**	279.5 [225.5-340.3]	181.0 [132.3-266.3]	.08
** Sclerostin (pg/mL)**	42.1 [26.3-74.4]	97.3 [46.7-154.4]	.07
** Dkk-1 (pg/mL)**	388.4 [258.2-495.6]	443.4 [303.5-597.7]	.49
** Wnt3a (ng/mL)**	6.2 [5.0–8.0]	4.2 [2.7-5.1]	.01
** Wnt3a/sclerostin**	0.16 [0.08–0.32]	0.04 [0.02-0.09]	.02
** Wnt3a/Dkk-1**	0.02 [0.01-0.02]	0.01 [0.01–0.02]	.03
** RANKL (pg/mL)**	220.5 [20.7-248.4]	137.1 [46.8-293.8]	>.99
** OPG (pg/mL)**	3384 [3028-4197]	2848 [2102-3309]	.06
** RANKL/OPG**	0.07 [0.01–0.01]	0.04 [0.02-0.11]	.77
**Urine pentosidine (pmol/mg Cr)**	18.3 [13.9-26.5]	10.0 [6.9–11.4]	<.0001
**BMD (g/cm** ^**2**^**)**			
** LS**	0.88 [0.79-1.07]	0.85 [0.82-1.00]	.60
** FN**	0.54 [0.43-0.62]	0.63 [0.59-0.72]	.03
** TH**	0.64 [0.45-0.75]	0.76 [0.73-0.84]	.01

Abbreviations: BAP, bone alkaline phosphatase; BP, bisphosphonate; eGFR, estimated glomerular filtration rate; NTX, type I collagen cross-linked N-telopeptide; OC, osteocalcin; OPG, osteoprotegerin; ROMO, romosozumab; TRACP-5b, tartrate-resistant acid phosphatase isoform-5b.

Data are expressed as the median with interquartile range.

### Changes in BMD

This study was analyzed using the FAS; consequently, 2 patients in the BP group who withdrew their consent by month 6 and had no subsequent data available were excluded from the analysis. There was no significant difference in cumulative annual GC dose between the 2 groups, but it tended to be higher in the ROMO group (median [25th**-**75th percentile]: ROMO group: 2442 [1755**-**3293] mg; BP group: 1823 [1820**-**2226] mg; *p* = .72).

**Figure 1:**
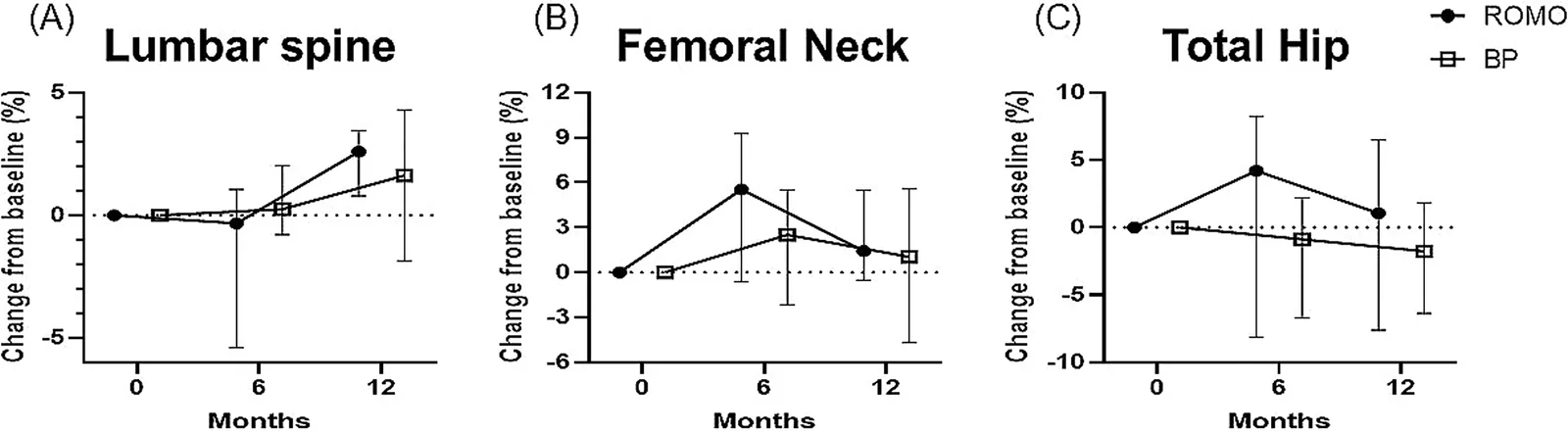
Percent changes in BMD from baseline in patients with glucocorticoid-induced osteoporosis in each treatment group of the LS (A), FN (B), and TH (C). Changes in the ROMO group (closed circles), and BP group (open quadrangles) from base line. Data are expressed as medians with interquartile ranges. Abbreviations: BP, bisphosphonate; ROMO, romosozumab.

Figure 1 shows the median percent change from baseline in BMD of the LS, FN and TH at 6 and 12 mo in each group. The median change in LS BMD [25**-**75th percentile] showed a slight decrease in the ROMO group at 6 mo (−0.3 [−5.4−1.1] %), but an increase at 12 mo (2.6 [0.8**-**3.4] %). In contrast, the BP group showed an increase at 6 mo (0.2 [−0.8−2.0] %) and at 12 mo (1.6 [−1.9−4.3] %) (Figure 1A). However, the percent change in LS BMD at both time points was not statistically significantly different from baseline in either group. The ROMO group exhibited a greater percentage increase in LS BMD at 12 mo compared to the BP group; however, this difference was not statistically significant.

The median change in FN BMD was 5.5 [−0.6−9.3] % at 6 mo and 1.4 [−0.5−5.4] % at 12 mo in the ROMO group. Increases were also observed in the BP group at 6 mo (2.5 [−2.1−5.5] %) and 12 mo (1.0 [−4.7−5.6] %), but no significant difference compared to baseline was observed, nor was there a significant difference between the 2 groups (Figure 1B). The median change in TH BMD increased at 6 mo (4.2 [−8.1−8.3] %) and at 12 mo (1.1 [−7.6−6.5] %) in the ROMO group. The BP group showed decreases at both time points (−0.9 [−6.7−2.2] % and −1.8 [−6.4−1.8] %, respectively) (Figure 1C). There were no significant differences from baseline or between the 2 groups at either time point. However, numerically, the ROMO group showed a greater percentage increase in BMD of the FN and TH at all time points compared to the BP group.

Given that the ROMO group was significantly older than the BP group, we compared the percent changes in BMD from baseline to 12 mo between the 2 groups using an age-adjusted linear regression model (Table 2). The ROMO group did not show significantly different percent changes in BMD from baseline to 12 mo compared with the BP group at any site, including the LS (*β* = .002, 95% CI −0.052 to 0.056, *p* = .941), FN (*β* = −.030, 95% CI −0.103 to 0.043, *p* = .427), and TH (*β* = .041, 95% CI −0.023 to 0.106, *p* = .222). Nonetheless, the percent changes in BMD of the LS and TH tended to be slightly greater in the ROMO group, while the FN showed a slightly smaller percent change in the ROMO group.

**Table 2:** Multivariable linear regression analyses for percentage change in BMD from baseline to 12 mo.

**Outcome**	**Variable**	**β**	**95% CI**	***p* value**
**LS**	ROMO vs BP	0.002	−0.052 to 0.056	.94
	Age	0.000	−0.001 to 0.002	.73
**FN**	ROMO vs BP	−0.030	−0.103 to 0.043	.43
	Age	0.002	0.000 to 0.004	.02
**TH**	ROMO vs BP	0.041	−0.023 to 0.106	.22
	Age	−0.001	−0.003 to 0.001	.25

### Bone turnover markers

**Figure 2:**
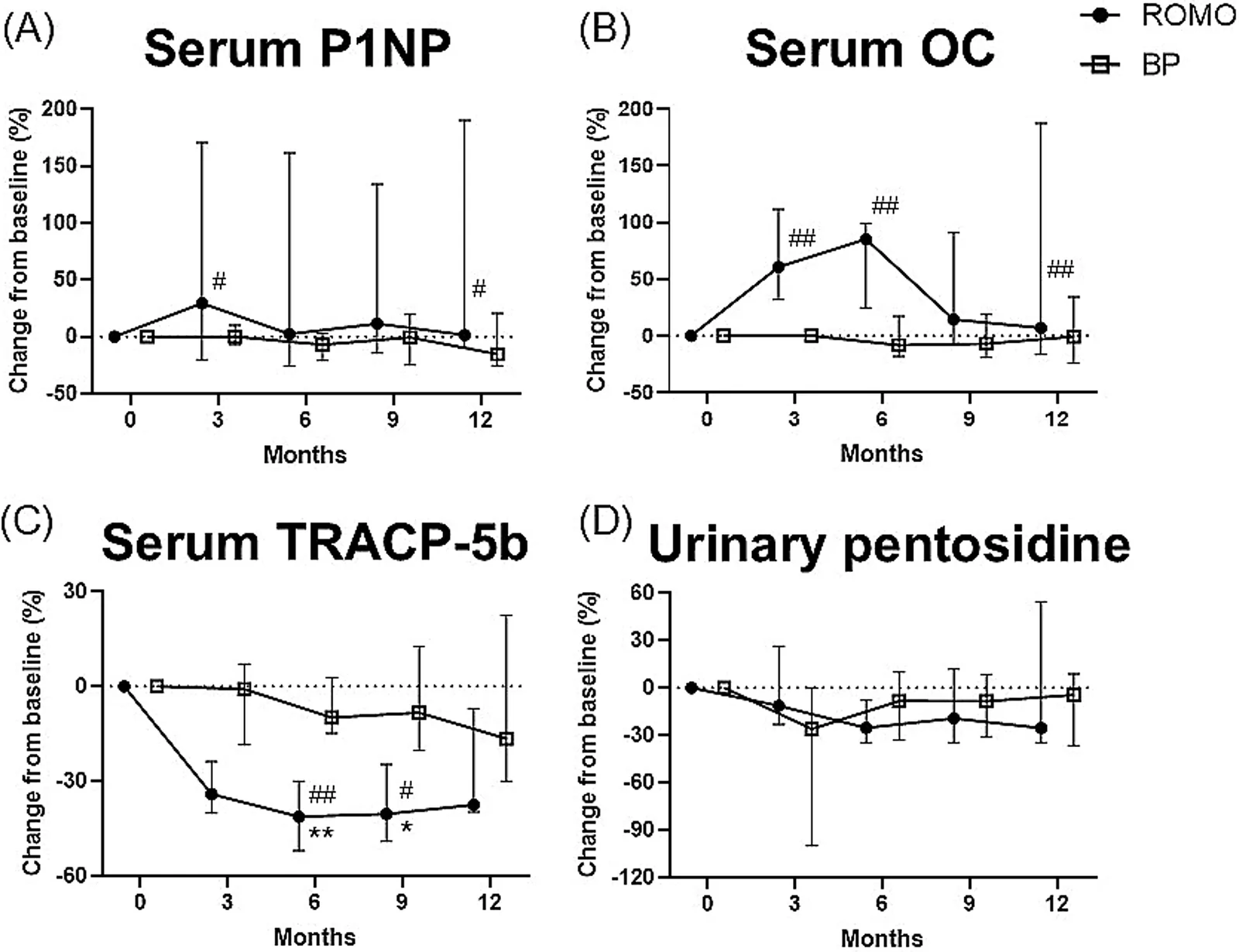
Percent changes from baseline in serum P1NP (A), OC (B), TRACP-5b (C), and urine pentosidine (D) levels in each treatment group of patients with GC-induced osteoporosis. Changes in the ROMO group (closed circles), and BP group (open quadrangles) from base line. Data are expressed as medians with interquartile ranges. ^*^p < .05, ^**^p < .01 vs the baseline within each treatment group by Dunn’s multiple comparison test. ^#^ROMO vs BP (^#^*p <* .05; ^##^*p <* .01) by Bonferroni’s multiple comparison test. Abbreviations: BP, bisphosphonate; OP, osteocalcin; ROMO, romosozumab; TRACP-5b, tartrate-resistant acid phosphatase 5b.

Serum levels of P1NP, a marker of bone formation, increased in the ROMO group at 3 mo but declined to approximately baseline values from 6 mo onward. In contrast, P1NP levels decreased in the BP group. The increase was significantly greater in the ROMO group compared to the BP group at 3 and 12 mo (Figure 2A). Serum levels of OC, another bone formation marker, increased in the ROMO group from 3 mo, but decreased from 9 mo onward. Conversely, the BP group showed no significant changes from 3 mo onward. The increase was significantly greater in the ROMO group compared to the BP group at 3, 6, and 12 mo (Figure 2B).

Serum levels of TRACP-5b, a bone resorption marker, decreased in both groups from 3 mo onward. Notably, the ROMO group demonstrated statistically significant reductions at 6 and 9 mo relative to baseline values and compared to the BP group (Figure 2C). Additionally, urinary pentosidine, a marker inversely associated with bone quality, decreased in both groups following the 3-mo period (Figure 2D). However, no significant differences were detected in percent change from baseline either within each group or between the 2 groups.

### Molecules regulating bone metabolism

**Figure 3:**
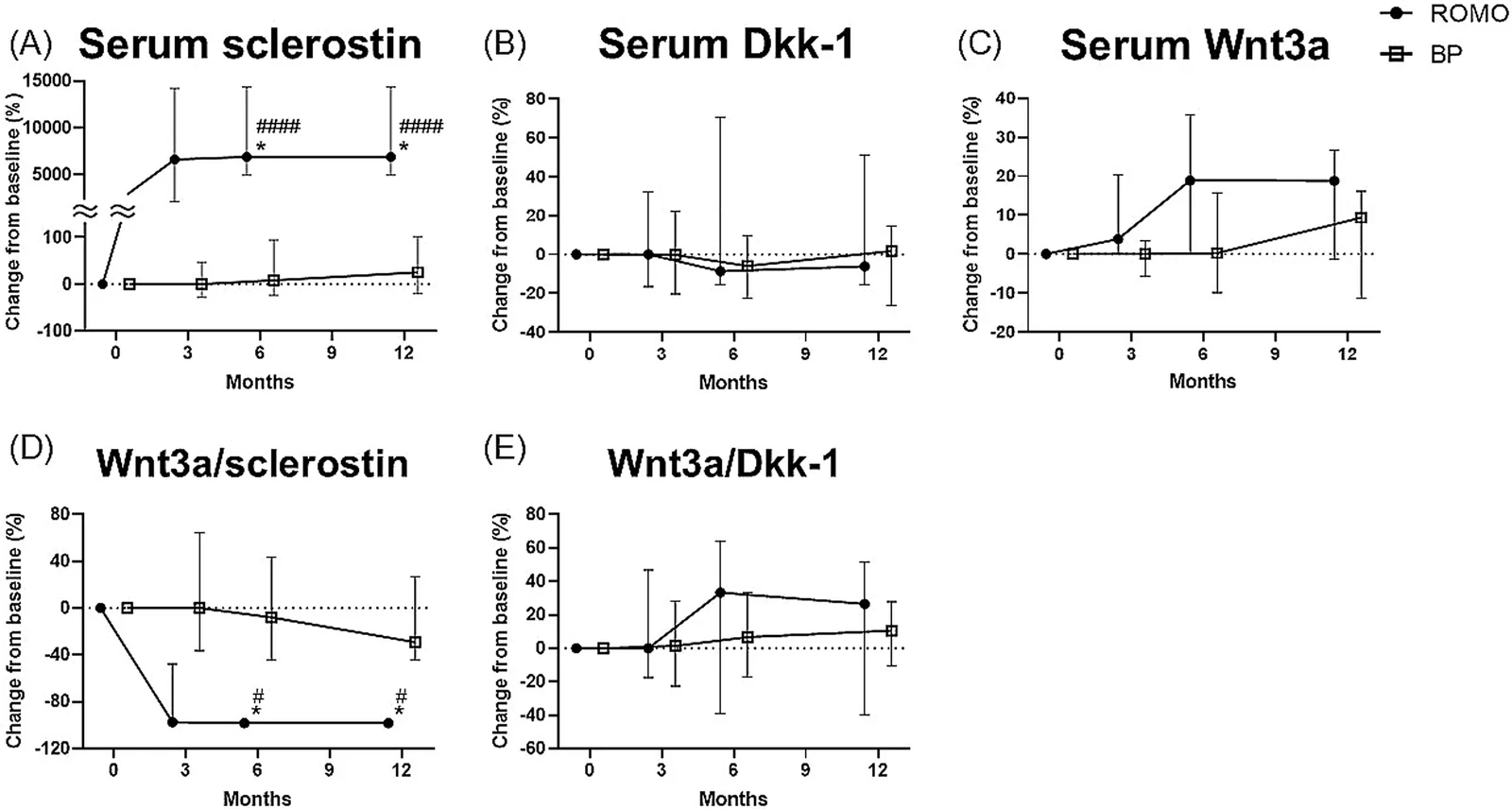
Percent changes from baseline in serum sclerostin (A), Dkk-1 (B), Wnt3a (C), Wnt3a/sclerostin (D), and Wnt3a/Dkk-1 (E) levels in each treatment group of patients with GC-induced osteoporosis. Changes in the ROMO group (closed circles), and BP group (open quadrangles) from base line. Data are expressed as medians with interquartile ranges. ^*^p < .05 vs the baseline within each treatment group by Dunn’s multiple comparison test. ^#^ROMO vs BP (^#^*p <* .05; ^####^*p <* .0001) by Bonferroni’s multiple comparison test. Abbreviations: BP, bisphosphonate; Dkk-1, Dickkopf-1; ROMO, romosozumab.

We assessed the levels of Wnt signaling inhibitors, sclerostin and Dkk-1, along with the Wnt signaling ligand Wnt3a. Serum sclerostin levels showed a significant increase in the ROMO group starting at 3 mo and remained elevated thereafter (Figure 3A). In the BP group, serum sclerostin levels increased gradually over time, but no significant difference was observed compared to baseline. Comparing the ROMO and BP groups, the rate of increase in the ROMO group was significantly higher at 6 and 12 mo. Serum Dkk-1 levels declined from 6 mo onward in the ROMO group, whereas in the BP group, they decreased at 6 mo but increased at 12 mo (Figure 3B). The rate of increase in each group, when compared to their respective baseline levels or between the 2 groups, showed no significant difference. Serum Wnt3a levels increased from 3 mo in the ROMO group and at 12 mo in the BP group; however, the increase did not reach statistically significance. Although the ROMO group exhibited a higher rate of increase compared to the BP group, the difference was not statistically significant (Figure 3C). The ratio of serum Wnt3a to sclerostin significantly decreased from 6 mo in the ROMO group, whereas no significant change was observed in the BP group. In the ROMO group, this ratio was significantly lower than that of the BP group from 6 mo (Figure 3D). The ratio of serum Wnt3a to Dkk-1 increased in both groups from 3 mo; however, it did not show a significant increase compared to baseline throughout the study period (Figure 3E).

**Figure 4:**
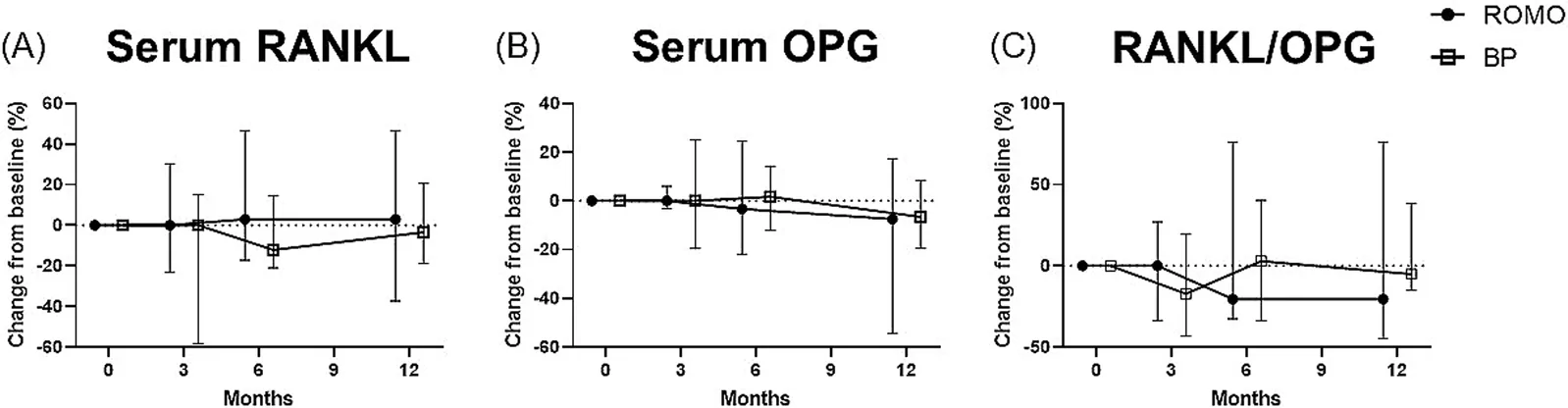
Percent changes from baseline in serum RANKL (A), OPG (B), and RANKL/OPG (C) levels in each treatment group of patients with GC-induced osteoporosis. Changes in the ROMO group (closed circles), and BP group (open quadrangles) from base line. Data are expressed as medians with interquartile ranges. Abbreviations: BP, bisphosphonate; OPG, osteoprotegerin; ROMO, romosozumab.

We also measured serum levels of RANKL, a molecule involved in osteoclast differentiation and maturation, and OPG, a RANKL inhibitor. Serum levels of RANKL increased slightly from 6 mo in the ROMO group, whereas they decreased from 6 mo in the BP group (Figure 4A). However, neither group showed statistically significant changes compared to baseline, and no significant differences were observed between the 2 groups at any time point. Regarding serum levels of OPG, a non-significant decline was observed from 6 mo in the ROMO group and at 12 mo in the BP group; however, no statistically significant differences were detected between the groups (Figure 4B). The ratio of serum RANKL to OPG decreased from 6 mo onward in the ROMO group. In contrast, the BP group showed a reduction at 3 mo, followed by a return to baseline levels thereafter (Figure 4C). These changes did not reach statistical significance when compared to baseline within each group or between groups.

### New fractures

During the 12-mo follow-up, 1 new vertebral fracture was observed only in the BP group. No non-vertebral fractures were reported in either group.

### Adverse events

A single adverse event was observed in each of the ROMO and BP groups. In the ROMO group, 1 patient—a 73-yr-old female diagnosed with vasculitis syndrome—experienced a cerebral infarction following the administration of 7 doses of ROMO, leading to discontinuation of the treatment; however, the causal association with ROMO is undetermined. In the BP group, 1 patient developed femoral head necrosis, with the causal relationship to the treatment also remaining unclear. No treatment-related deaths were observed.

## Discussion

This study demonstrated that in patients receiving long-term oral GC and BP therapy, switching to ROMO for 12 mo resulted in greater increases in the BMD of the LS, FN, and TH compared to continuing BP. Even after adjusting for age, the ROMO group still exhibited a slight tendency toward a greater increase in the BMD of the LS and TH compared to the group that continued BP. These findings suggest that ROMO may be an effective sequential therapy for patients undergoing BP treatment in the context of GIOP.

To our knowledge, no previous studies have directly compared the efficacy of continuing BP therapy vs switching to ROMO for treating osteoporosis in patients receiving both GC and BP therapy. However, a RCT has reported the therapeutic effects of ROMO compared to denosumab (DMAb), an anti-RANKL antibody, in patients receiving long-term low-dose PSL (median dose 5.0 mg/d; treatment duration: mean ± SE, 122.3 ± 89 mo).^11^ In subgroup analysis, patients receiving ROMO with prior BP therapy showed a mean increase in LS BMD of 5.2% and FN BMD of 0.7% at 12 mo. In contrast, patients without prior BP therapy exhibited a 10% increase in LS BMD and a 2.8% increase in FN BMD, indicating a higher rate of improvement.

Similarly, in our previous retrospective study investigating the effectiveness of DMAb in patients with rheumatic diseases (68.5% using GCs; median PSL-equivalent dose 5.0 mg/d), patients with prior BP use exhibited a lower median percentage increase in LS BMD at 12 mo compared to those without prior BP use (3.6% vs 4.1%).^12^ Furthermore, patients receiving GCs at doses equivalent to 5 mg or more showed a lower median percentage increase in LS BMD at 12 mo compared to those not using GCs (2.8% vs 5.5%). Further research is needed to compare the efficacy of ROMO and DMAb in GIOP patients with prior BP use.

A case-control study comparing the effects of switching from BP therapy to ROMO, DMAb or teriparatide in patients with PMO reported a mean increases in LS BMD of 11.4%, FN BMD of 2.0% and TH BMD of 3.3% at 12 mo in the patients in the ROMO group.^13^ Our previous RomGo study evaluated the therapeutic effects of ROMO, DMAb, and BP in patients with rheumatic diseases who had no history of osteoporosis treatment and were initiating moderate to high doses of GCs (median 20.0 mg/d).^10^ After 12 mo, the median changes in BMD in the ROMO group were 8.6% of the LS, 0.5% of the FN and 2.1% of the TH. These results suggest that the BMD-increasing effect of ROMO may be diminished in patients who have previously received BP therapy, or long-term GC therapy even at low doses.

In this study, serum P1NP levels increased in the ROMO group at 3 mo and returned to baseline after 6 mo. Similar changes have been reported in the aforementioned RCT involving patients receiving low-dose GCs.^11^ In the ARCH study of postmenopausal women with osteoporosis, serum P1NP increased at 1 mo after ROMO administration, then gradually decreased, returning to baseline levels after 9 mo.^8^ In contrast, in the RomGo study, patients were initiated on moderate to high doses of GCs, and serum P1NP levels were below baseline from 3 mo.^10^ These results suggest that under long-term, low-dose GC treatment, the bone formation-promoting effect of ROMO outweighs the bone formation-suppressing effect of GCs. In this study, serum P1NP levels increased at 3 mo but decreased after 6 mo—earlier than in postmenopausal women—likely due to the effects of GCs. While serum P1NP is considered an early marker of bone formation, serum OC is regarded as a later marker because it is produced by mature osteoblasts.^14^ Although GCs are known to inhibit bone turnover and reduce OC levels, serum OC increased after ROMO administration in this study and, unlike P1NP, remained elevated for up to 6 mo. In the RomGo study, OC levels were also below baseline from 3 mo.^10^ The suppressive effect on bone formation appears to be stronger during the initiation of moderate to high doses of GCs compared to the use of long-term, low-dose GCs, under treated with ROMO.

The decrease in serum levels of TRACP-5b was greater in the ROMO group than in the BP group in this study. In the previously mentioned RCT involving patients on low-dose GC, serum C-terminal cross-linked telopeptide collagen, another marker of bone resorption, was measured, and it began to decrease 6 mo after the start of ROMO.^11^ Therefore, it was suggested that ROMO may exert a strong inhibitory effect on bone resorption, even in patients undergoing long-term GC administration and BP therapy.

Baseline levels of urinary pentosidine, which is thought to inversely reflect bone quality,^14^ were significantly higher in the ROMO group than in the BP group. This difference is likely attributable to the ROMO group consisting of elderly patients and a higher proportion of individuals with a history of fracture prior to the commencement of the study. After the start of the study, the urine levels decreased in both groups, but no significant differences were observed. Therefore, both ROMO and BP may slightly improve bone quality in GIOP.

Serum levels of sclerostin were markedly elevated in the ROMO group, consistent with our RomGo study.^10^ This increase is hypothesized to result from the prolonged elimination half-life of sclerostin due to its complex formation with ROMO. ROMO is a high-affinity antibody against sclerostin, and a similar mechanism is thought to apply here, as serum sclerostin levels increased in a dose-dependent manner following ROMO administration in the phase I study.^15^ Consequently, it is assumed that free sclerostin levels decreased in the ROMO group. Incidentally, GCs have been shown to suppress Wnt signaling and reduce bone formation by increasing the production of Wnt signaling inhibitors, such as sclerostin and Dkk-1, while decreasing ligands like Wnt3a.^16^^,^^17^ In the ROMO group, both free sclerostin and Dkk-1 levels decreased, indicating a reduction in Wnt signaling inhibitors, whereas the ligand Wnt3a increased. Furthermore, the Wnt3a/Dkk-1 ratio, representing the ligand-to-inhibitor ratio, increased, suggesting that Wnt signaling may be activated, thereby promoting bone formation. Conversely, in the BP group, although sclerostin and Dkk-1 levels increased, Wnt3a also increased. The Wnt3a/Dkk-1 ratio increased, but the Wnt3a/sclerostin ratio decreased, indicating that while Wnt signaling-mediated promotion of bone formation was observed, it was less pronounced than in the ROMO group.

No significant changes were observed in serum levels of RANKL and OPG in either group, but the rate of decrease in RANKL/OPG was greater in the ROMO group than in the BP group, suggesting that increased bone resorption due to promotion of osteoclast differentiation and maturation may have been more suppressed in the ROMO group.

Regarding adverse events, 1 patient in the ROMO group experienced a cerebral infarction. Because the patient was elderly and had vasculitis syndrome, the risk of cerebrovascular events was thought to be high, and the causal relationship with ROMO is unclear. Further studies are needed to establish the cerebrovascular and cardiovascular safety of ROMO in patients receiving long-term GCs.

This study has several limitations. First, it was an observational study, which may have led to selection bias. Second, due to the COVID-19 pandemic, some patients refused to receive ROMO, which requires monthly hospital visits. This hindered patient enrollment as planned, resulting in a small sample size. Third, some baseline characteristics differed between the ROMO and BP groups, including age, history of vertebral fractures, and BMD of the FN and TH. Due to the small number of patients analyzed, we were unable to adjust for variables other than age. These factors may have influenced the results. Furthermore, the limited number of patients and the short observation period make it difficult to conduct a comparative assessment of fracture prevention effects and to adequately evaluate safety issue. Therefore, future large-scale, long-term studies are needed to clarify the effects of ROMO administration on BMD increase and fracture prevention.

In conclusion, this study demonstrated that the sequential use of ROMO increased BMD of the LS, FN and TH compared to continuous administration of BP in patients with GIOP who had long-term BP exposure. However, the differences did not reach statistical significance. Future studies with longer durations and larger patient populations are warranted.

## Data Availability

The data underlying this article will be shared on reasonable request to the corresponding author.
